# Human Health Risk Assessment From Exposure to Toxic Metals in Soils and Leafy Vegetables in Smallholder Farms of Northeastern Brazil

**DOI:** 10.1002/jat.70164

**Published:** 2026-03-19

**Authors:** Fernando Ferreira da Cunha Filho, Alex Souza Moraes, João Paulo Siqueira da Silva, Edvane Borges da Silva, Ademir Amaral, André Maciel Netto

**Affiliations:** ^1^ Instituto Federal de Educação, Ciência e Tecnologia de Pernambuco (IFPE), Campus Belo Jardim Pernambuco Brazil; ^2^ Department of Chemistry Federal Rural University of Pernambuco Recife Pernambuco Brazil; ^3^ Pernambuco Department of Education Recife Pernambuco Brazil; ^4^ Academic Center of Vitória Federal University of Pernambuco Vitoria de Santo Antão Pernambuco Brazil; ^5^ Department of Nuclear Energy Federal University of Pernambuco Recife Pernambuco Brazil

**Keywords:** human health risk assessment, leafy vegetables, smallholder farming, soil ingestion, toxic metals

## Abstract

This study assessed human health risks associated with exposure to potentially toxic metals (PTMs) in soils and leafy vegetables cultivated in smallholder agricultural systems in Northeastern Brazil. Soil and vegetable samples were collected from 18 agricultural areas and analyzed for cadmium (Cd), chromium (Cr), copper (Cu), iron (Fe), nickel (Ni), lead (Pb), and zinc (Zn). A harmonized human health risk assessment (HHRA) was conducted following US Environmental Protection Agency (USEPA) guidelines, considering soil ingestion, dermal contact, inhalation of resuspended particles, and dietary intake of vegetables for adults and children. Recalculated average daily doses indicated that soil ingestion and vegetable consumption were the dominant exposure pathways, whereas dermal and inhalation contributions were minor. Children consistently exhibited higher exposure doses and hazard quotients than adults due to lower body weight and higher intake rates. Noncarcinogenic risk estimates for adults remained below the threshold of concern in all areas. In contrast, localized exceedances of the hazard index (*HI* > 1) were identified for children, revealing spatially explicit risk hotspots rather than uniform risk across the study area. Lettuce and arugula contributed substantially to dietary exposure, particularly for Cd, Pb, and Cr. A conceptual sensitivity analysis incorporating hypothetical bioaccessibility fractions demonstrated that risk estimates near the regulatory threshold are sensitive to realistic reductions in bioaccessible metal fractions. At the same time, the highest risk hotspots remain robust under conservative assumptions. Overall, the findings represent conservative, screening‐level estimates that underscore the importance of age‐specific exposure scenarios, spatially explicit risk evaluation, and targeted risk management strategies in smallholder farming systems.

## Abstract 2

This study provides a harmonized human health risk assessment of exposure to potentially toxic metals in soils and leafy vegetables from smallholder farming systems in Northeastern Brazil. Using USEPA‐consistent exposure factors, noncarcinogenic risks were evaluated for adults and children through soil ingestion, dermal contact, inhalation, and dietary intake. Results indicate that soil ingestion and vegetable consumption dominate exposure pathways, with children showing higher vulnerability and localized risk hotspots, highlighting the importance of age‐specific and spatially explicit risk assessment.

## 1 Introduction

Soil contamination by potentially toxic metals (PTMs) represents a persistent global challenge for environmental health, particularly in agricultural systems subjected to long‐term anthropogenic pressure. Intensive farming practices, including the excessive use of mineral fertilizers, organic amendments, and pesticides, have been widely recognized as major contributors to the accumulation of metals such as cadmium (Cd), lead (Pb), chromium (Cr), nickel (Ni), copper (Cu), and zinc (Zn) in cultivated soils (Münzel et al. 2023; Rashid et al. 2023; Angon et al. 2024; Cai 2025). Unlike organic contaminants, these elements are nonbiodegradable and can persist in soils for decades, increasing the likelihood of chronic human exposure through multiple environmental pathways.

Human exposure to PTMs in agricultural landscapes occurs predominantly through the ingestion of contaminated food, but dermal contact and inhalation of resuspended soil particles may also represent relevant exposure routes, especially in rural and peri‐urban settings (Khan et al. 2019; Baltas et al. 2020). Children are particularly vulnerable due to behavioral factors and higher intake rates per unit body weight (BW), relative to adults, which collectively amplify noncarcinogenic health risks (Cabral‐Pinto et al. 2020; Ahmad et al. 2021). Consequently, human health risk assessment (HHRA) has become an essential tool for translating environmental concentration data into toxicologically meaningful metrics that support public health protection and regulatory decision‐making.

Most HHRA studies addressing PTM contamination in soils and food crops have focused on industrialized regions or temperate agricultural systems, often under well‐regulated management practices (Tepanosyan et al. 2017; Yang, Liu, et al. 2019). In contrast, smallholder farming systems in low‐ and middle‐income countries remain underrepresented in the toxicological literature, despite their central role in local food security and their frequent reliance on poorly regulated agricultural inputs. In tropical regions, continuous cultivation, high rates of organic matter turnover, and intensive fertilizer application can further modify metal mobility and bioavailability, potentially exacerbating human exposure risks (Hu et al. 2017; Paltseva et al. 2018).

Brazil exemplifies this scenario. Peri‐urban horticulture is widely practiced under limited technical guidance, and leafy vegetables—such as lettuce and arugula—are cultivated year‐round to meet local demand. These crops are particularly prone to metal accumulation due to their shallow root systems, rapid growth cycles, and high consumption rates (CRs) (Berton 2000; Azeez et al. 2020). While several studies have reported elevated PTM concentrations in Brazilian agricultural soils and vegetables, fewer have rigorously integrated these data into US Environmental Protection Agency (USEPA)–based HHRA frameworks that account for multiple exposure pathways and vulnerable populations (Baltas et al. 2020; Perković et al. 2022).

An additional limitation in existing literature concerns methodological inconsistencies in HHRA applications, including the use of non‐harmonized exposure parameters, inappropriate reference doses across exposure routes, and the frequent neglect of food consumption on a fresh‐weight basis. Such issues may lead to either underestimation or overestimation of health risks, thereby compromising the reliability of risk‐based recommendations (USEPA 2011, 2019). Addressing these gaps requires both quantitative recalculation and conceptual refinement of HHRA approaches, particularly in studies intended to inform public health and food safety policies.

In this context, the present study conducts a comprehensive reassessment of human health risks associated with exposure to PTMs in soils and leafy vegetables cultivated in smallholder farming areas of Northeastern Brazil. By integrating validated analytical chemistry methods with a fully harmonized USEPA HHRA framework, this work evaluates noncarcinogenic risks for adults and children through soil ingestion, dermal contact, inhalation, and dietary intake of vegetables. Beyond site‐specific risk characterization, the study provides a defensible and conservative toxicological assessment that highlights the implications of intensive smallholder agriculture for food safety and human health in tropical environments.

## 2 Material and Methods

### 2.1 Study Area

The study was conducted in Vitória de Santo Antão, located in the Mata Sul mesoregion of Pernambuco State, Brazil, which is the largest producer of leafy vegetables in the state. The region has a tropical humid climate with dry summers, average annual temperatures ranging from 19.8°C to 34.9°C, and average annual precipitation of 1309.9 mm, with October being the driest month (24 mm) and June the wettest (158 mm) (Instituto Nacional de Meteorologia [INMET] 2024). Geologically, it is situated in the Borborema Province, consisting of lithotypes from the Belém do São Francisco Complex (gneisses and migmatites) (Figure 1). Soils are predominantly Haplic Luvisols (Ferric, Eutric, Humic), deep and well‐drained, according to the World Reference Base for Soil Resources (2022).

**FIGURE 1:**
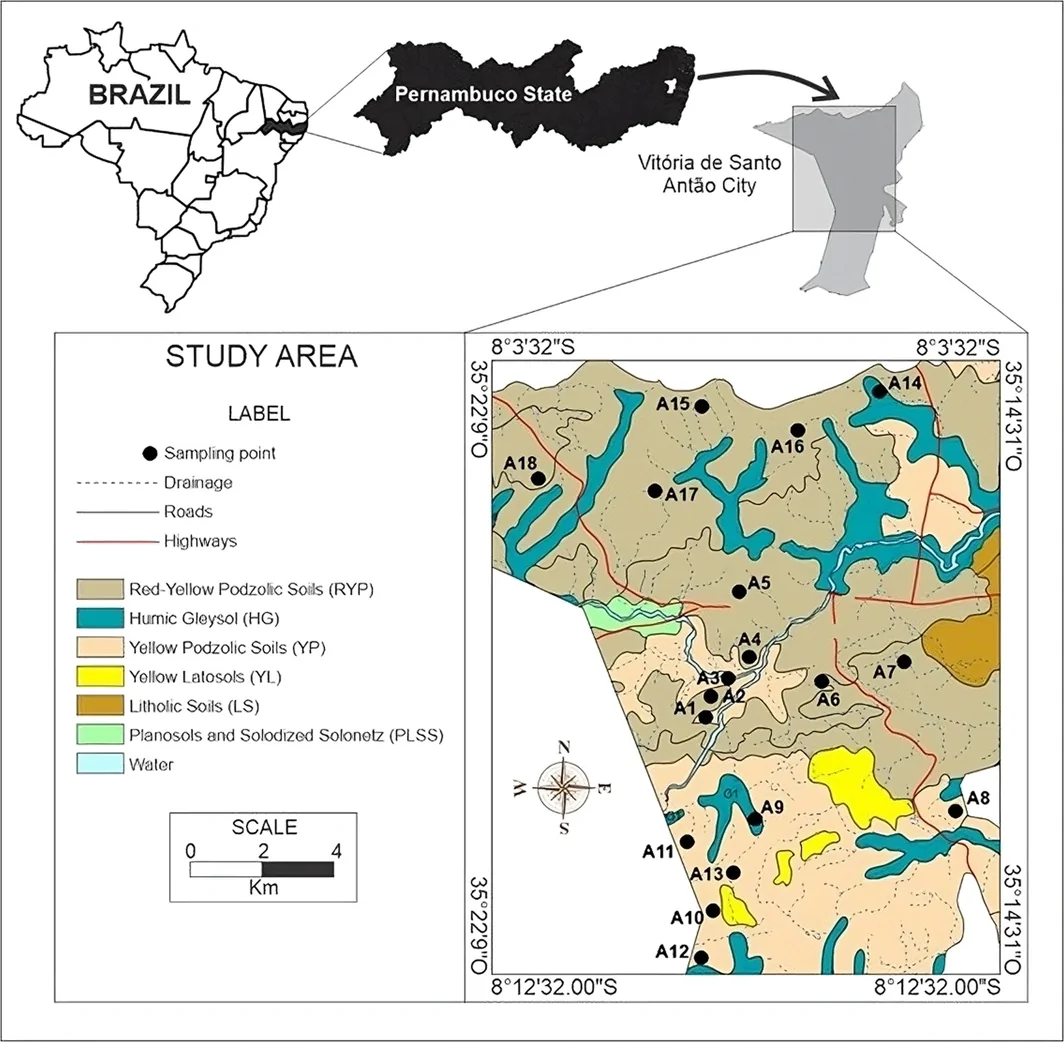
Location of collection points in the city of Vitória de Santo Antão‐PE. *Source:* The black dots indicate the location of the study areas.

The geographic location, coordinates, and identification of the 18 smallholder agricultural areas are provided in Table S1 (Supporting Information).

Natuba, the main producing area in the metropolitan region of Vitoria de Santo Antão, was subdivided into subareas based on factors that could contribute to environmental contamination. Farmers perform all activities, from planting to preparing vegetables for sale, adopting similar soil management practices regardless of crop type or soil class, using raised beds 0.20 to 0.40 m high and of variable length. Information on cultivated area size, dominant crops, and cultivation history for each sampling site is summarized in Table S2 (Supporting Information).

A total of 18 vegetable‐producing sites were selected based on their agricultural importance and environmental vulnerability. These smallholder agricultural areas were georeferenced using GPS, and data on cultivation history, fertilization practices, and land management were collected through interviews with farmers and field observations.

### 2.2 Soil Sampling and Sample Preparation

Soil samples were collected from each production bed at a depth of 0–20 cm in March 2016. At each of the 18 sites, three composite samples were formed from 10 subsamples each, for a total of 54 soil samples. Sampling was performed using stainless steel augers to prevent contamination. Samples were stored in polyethylene bags and transported to the Federal University of Pernambuco (UFPE), where they were air‐dried, sieved (2 mm), and stored for chemical and physical analyses.

### 2.3 Collection of Plant Samples

Vegetable samples were also collected in the soil collection areas. In areas A1, A2, and A4, whole plant specimens (roots, stems, leaves, flowers, and fruits when present in the crop) were collected (Supporting Information, Table S4), while in the remaining areas, only the edible parts of lettuce ( *Lactuca sativa* L., cultivar Vitória) were sampled. After field collection, plant materials were washed with tap water, distilled water, mild detergent (0.3%), and ultrapure water. Samples were oven‐dried at 65°C to constant weight, ground in a Willey mill (20 mesh), and stored in plastic bags for metal analysis. The number of samples per crop varied according to the type and quantity available for supply. Details on the collected crops, analyzed plant organs, production characteristics, and corresponding sampling areas are reported in Table S4 (Supporting Information).

### 2.4 Agricultural Inputs

Details on fertilizers, pesticides, and organic amendments applied in the studied areas, including application frequency and doses, are presented in Table S3 (Supporting Information).

Farmers provided samples of fertilizers and pesticides. Granular inputs were macerated in an agate mortar, sieved (0.3 mm), and stored in plastic jars. Organic inputs (cattle manure and chicken litter) were randomly sampled from storage areas, forming composite samples for each input type. To determine the moisture, the material remained in an oven at 65°C until it reached constant weight. The dried samples were then ground in a Willey‐type mill, equipped with a 20‐mesh stainless steel sieve.

### 2.5 Chemical and Physical Analyses of Soils

Soil pH, exchangeable bases (Ca^2+^, Mg^2+^, K^+^, Na^+^), aluminum (Al^3+^), potential acidity (H^+^+Al^3+^), organic carbon (OC), total organic matter (OM), available phosphorus (P), and cation exchange capacity (CEC) were determined following standard methods recommended by EMBRAPA (Teixeira et al. 2017). The physical and chemical attributes used as input parameters for HHRA are presented in Table S5 in the Supporting Information.

### 2.6 Metal Analysis in Soils, Plants, and Inputs

The concentrations of Cd, Cr, Cu, Fe, Ni, Pb, and Zn were determined using microwave‐assisted acid digestion (USEPA Method 3051A, USEPA 2007). Soil and organic input samples (1 g) were digested with 9 mL of HNO_3_ (65%) and 3 mL of HCl (37%). Plant samples (0.5 g) underwent identical digestion. Element concentrations were quantified using flame atomic absorption spectrometry (FAAS) for Fe, Cu, and Zn, and graphite furnace AAS (GFAAS) for Cd, Cr, Ni, and Pb, with quality assurance conducted using certified reference materials (NIST SRM 2711a and SRM 1570a) (National Institute of Standards and Technology, 2003, 2015). The chemical composition of organic fertilizers (cattle manure and chicken litter) applied in the study area is reported in Table S6 (Supporting Information).

### 2.7 Metal Transfer and Translocation in Plants

Metal uptake was evaluated using the transfer factor (*t*) (Supporting Information, Table S10) and the metal translocation indices from roots to edible parts (*TI*) (Supporting Information, Table S11), which quantify the absorption and movement of metals within the plants, according to equation (1):

(1) $$t=\frac{\text{Metal in the plant}}{\text{Metal in the soil}}$$

The total metal content of the plant was calculated by summing the metal contents of the shoot (MSPA), roots (MSR), stem (MSC), flower (MSF), and fruit (MSFr), when available in the crop. The higher the *t*‐factor, the greater the absorption of the metal (Ali et al. 2013; Hesami et al. 2018; Ashraf et al. 2019).

Through the translocation index (*TI*), it is possible to evaluate the ability of vegetables to translocate metals from the root to the aerial part. The *TI* is calculated by Equation (2) (Paiva et al. 2002; Su et al. 2019):

(2) $$\mathrm{TI}\;\text{aerial part}=\frac{\text{MSPA content}}{\left(\mathrm{MSR}+\text{MSPA}\right)\text{content}}$$

### 2.8 HHRA

The HHRA was conducted in strict accordance with the guidelines and methodological framework established by the USEPA, following the Risk Assessment Guidance for Superfund (RAGS), the Exposure Factors Handbook, and the Integrated Risk Information System (IRIS) (USEPA 1989, 2011, 2019; Barrio‐Parra et al. 2024). All exposure parameters previously derived from non‐harmonized sources were replaced with USEPA‐recommended values to ensure methodological consistency, toxicological relevance, and comparability with recent studies.

Noncarcinogenic risks associated with exposure to PTMs were evaluated for two population groups: adults and children. The assessment considered multiple exposure pathways, including incidental soil ingestion, dermal contact with contaminated soil, inhalation of resuspended soil particles, and dietary intake of leafy vegetables.

#### 2.8.1 Exposure Parameters

Population‐specific exposure parameters were selected to represent conservative yet realistic scenarios for the Brazilian population. Body weight (*BW*) was set at 70 kg for adults and 15 kg for children, conservative values aligned with the USEPA exposure recommendations and compatible with Brazilian anthropometric data. The exposure frequency (*EF*) was fixed at 350 days·year^−1^ for all exposure routes and population groups, reflecting chronic exposure conditions in agricultural environments. Exposure duration (*ED*) was assumed to be 24 years for adults and 6 years for children, while the averaging time (*AT*) for noncarcinogenic effects was calculated as *ED* × 350 days.

The exposed skin surface area (*SA*) was corrected according to USEPA guidelines and set at 5700 cm^2^ for adults and 2800 cm^2^ for children. Dermal adherence factor (*AF*), dermal absorption fraction (*ABS*), inhalation rate (*InhR*), and particle emission factor (*PEF*) were adopted from the USEPA Exposure Factors Handbook (USEPA 2011) as default values, given the lack of Brazil‐specific exposure factor data, providing a conservative and harmonized basis for risk estimation.

#### 2.8.2 Soil Exposure Assessment

##### 2.8.2.1 Soil Ingestion

The average daily dose (*ADD*_ing_, mg·kg^−1^·day^−1^) resulting from incidental soil ingestion was calculated using Equation (3):

(3) $${\mathrm{ADD}}_{\mathrm{ing}}=\frac{C_{s}\times {\mathrm{IR}}_{\text{soil}}\times \mathrm{EF}\times \mathrm{ED}}{\mathrm{BW}\times \mathrm{AT}}$$

where $C_{s}$ is the metal concentration in soil (mg·kg^−1^), $IR_{\text{soil}}$ is the soil ingestion rate (USEPA 2011), EF is the exposure frequency (350 days·year^−1^), ED is the exposure duration (years), BW is the body weight (kg), and AT is the averaging time (days). Calculated metal doses via inhalation of resuspended soil particles for adults and children are summarized in Table S12 (Supporting Information).

##### 2.8.2.2 Dermal Contact With Soil

The absorbed dose through dermal contact (*ADD*_*derm*_, mg·kg^−1^·day^−1^) was estimated according to Equation (4):

(4) $${\mathrm{ADD}}_{\text{derm}}=\frac{C_{s}\times \mathrm{SA}\times \mathrm{AF}\times \mathrm{ABS}\times \mathrm{EF}\times \mathrm{ED}}{\mathrm{BW}\times \mathrm{AT}}$$

where SA is the exposed skin surface area (cm^2^), AF is the soil‐to‐skin adherence factor (mg·cm^−2^), and ABS is the dermal absorption fraction, all adopted from USEPA recommendations. Estimated metal doses resulting from dermal contact with contaminated soil are provided in Table S13 (Supporting Information).

##### 2.8.2.3 Inhalation of Soil Particles

The inhalation exposure was calculated using a dimensionally consistent approach, with the daily exposure concentration expressed in mg·m^−3^, compatible with the inhalation reference concentration (RfCi). The exposure concentration (EC, mg·m^−3^) was estimated using Equation (5):

(5) $$\mathrm{EC}=\frac{C_{s}\times \text{InhR}\times \mathrm{EF}\times \mathrm{ED}}{\mathrm{PET}\times \mathrm{AT}}$$

where InhR is the inhalation rate (m^3^·day^−1^) and PEF is the particle emission factor (1.36 × 10^9^ m^3^·kg^−1^, US Department of Agriculture 2024), as defined by USEPA (2011).

#### 2.8.3 Dietary Exposure via Vegetable Consumption

Dietary exposure to PTMs through vegetable consumption was assessed by calculating the chronic daily intake (*CDI*, mg·kg^−1^·day^−1^), as shown in Equation (6):

(6) $$\mathrm{CDI}=\frac{C_{v}\times {\mathrm{IR}}_{\mathrm{veg}}\times \mathrm{EF}\times \mathrm{ED}}{\mathrm{BW}\times \mathrm{AT}}$$

where *C*_*v*_ is the metal concentration in vegetables expressed on a fresh‐weight basis (mg·kg^−1^), ensuring consistency with food ingestion rates, and $IR_{\mathrm{veg}}$ is the vegetable ingestion rate for adults (0.13 kg day^−1^ for lettuce and cucumber, 0.09 kg day^−1^ for arugula and radish and 0.019 kg day^−1^ for chives and mustard) (IBGE 2011) and for children, in which one third of an adult's consumption was adopted (Guerra et al. 2012), adopted from national dietary surveys and harmonized with USEPA exposure assumptions.

Metal concentrations in vegetables were originally determined on a dry‐weight basis following sample dehydration. To ensure dimensional consistency with dietary ingestion rates, which are expressed on a fresh‐weight basis, all concentrations were converted to fresh weight prior to risk calculations. This conversion was performed using crop‐specific moisture content values obtained from standardized food composition databases provided by FAO and INFOODS (2017) and the USDA FoodData Central (US Department of Agriculture 2024). Fresh‐weight concentrations were calculated by multiplying dry‐weight concentrations by the corresponding dry matter fraction of each crop, as expressed by the relationship $C_{\text{fresh}}=C_{\mathrm{dry}}\times \left(1-\mathrm{MC}\right)$, where MC represents the moisture content expressed as a fraction. This procedure avoids overestimation of dietary exposure and is consistent with USEPA recommendations for HHRA.

#### 2.8.4 Risk Characterization

The noncarcinogenic hazard quotient (*HQ*) for each metal and exposure route was calculated by dividing the estimated dose by the corresponding reference value, as shown in Equation (7):

(7) $$\mathrm{HQ}=\frac{\mathrm{ADD}\;\text{or}\;\mathrm{CDI}}{\mathrm{RfD}\;\text{or}\;\mathrm{Rf}c_{i}}$$

Route‐specific reference values were adopted as follows:

- Oral ingestion: *RfD*_ing_ (mg·kg^−1^·day^−1^)
- Dermal exposure: *RfD*_derm_ (mg·kg^−1^·day^−1^),
- Inhalation exposure: *RfC*_*i*_ (mg·m^−3^)

All reference values were obtained from the USEPA IRIS database (US Department of Agriculture 2024). For chromium, a conservative toxicological approach was adopted by avoiding reference values specific to insoluble Cr(III) salts and instead applying USEPA‐recommended values representative of total chromium exposure. Available CRs for vegetables, derived from oral reference doses and measured metal concentrations, are presented in Table S14 (Supporting Information).

The hazard index (*HI*) was calculated as the sum of *HQ* values across all exposure pathways and metals, as shown in Equation (8):

(8) $$\mathrm{HI}=\sum {\mathrm{HQ}}_{i}$$

An *HI* value greater than unity (*HI* > 1) was interpreted as indicative of a potential noncarcinogenic health concern.

When applicable, the safe consumption rate (*CR*, g·day^−1^) (Equation 9) for vegetables was estimated from the relationship between oral reference doses, *BW*, and measured metal concentrations, thereby providing an additional metric for dietary risk management.

(9) $$\mathrm{CR}=\frac{\mathrm{RfD}\times \mathrm{BW}}{C}$$

where *RfD* is the reference oral dose (mg kg^−1^ day^−1^); *BW* is the body mass and *C* is the concentration of the evaluated metal.

## 3 Results and Discussion

### 3.1 Soil Metal Concentrations and Exposure‐Relevant Context

The concentrations of PTMs measured in agricultural soils from Vitória de Santo Antão (Supporting Information, Table S7) revealed widespread enrichment of Cd, Pb, Ni, Cu, and Zn relative to Brazilian prevention and investigation values. Estimated cumulative inputs of metals from fertilizers and pesticides over a 10‐year cultivation period are detailed in Table S8 (Supporting Information), while metal contents in crop protection products are shown in Table S9 (Supporting Information). Although concentration‐based exceedances provide an initial indication of contamination, recent toxicological studies emphasize that human health risk cannot be reliably inferred from concentration data alone, particularly in agricultural systems where exposure pathways and bioaccessibility strongly modulate toxicological relevance (Tepanosyan et al. 2017; Münzel et al. 2023).

When translated into exposure doses using the harmonized USEPA framework, soil ingestion emerged as the dominant exposure pathway for both adults and children, followed by dermal contact and inhalation of resuspended particles. This hierarchy is consistent with findings reported in studies evaluating agricultural and peri‐urban soils, in which ingestion typically accounts for more than 70% of total noncarcinogenic risk (Baltas et al. 2020; Ahmad et al. 2021).

Table 1 shows that soil ingestion is the dominant exposure pathway for all metals in both adults and children, with ingestion doses exceeding those from dermal and inhalation exposure by several orders of magnitude. Iron presents the highest ingestion‐related doses, followed by zinc and copper, whereas dermal and inhalation contributions remain marginal. Children consistently exhibit higher *ADD* values than adults due to lower *BW* and higher intake rates, which are partly attributable to age‐related hand‐to‐mouth behavior leading to direct soil ingestion or indirect ingestion via house dust. These results highlight the predominance of soil ingestion and the greater vulnerability of children in soil exposure scenarios.

**TABLE 1:** Recalculated average daily doses (*ADDs*, mg·kg^−1^·day^−1^) for soil exposure pathways in adults and children.

Metal	Population	Ingestion—*ADD*_*ing*_ (mg·kg^−1^·day^−1^)	Dermal—*ADD*_*derm*_ (mg·kg^−1^·day^−1^)	Inhalation—*EC* (mg·m^−3^)
Cd	Adult	8.62E−06	3.44E−08	8.88E−08
Cr	Adult	4.82E−05	1.93E−07	4.97E−07
Cu	Adult	1.23E−04	4.91E−07	1.27E−06
Fe	Adult	1.78E−02	7.09E−05	1.83E−04
Ni	Adult	2.70E−05	1.08E−07	2.78E−07
Pb	Adult	6.63E−05	2.64E−07	6.82E−07
Zn	Adult	3.92E−04	1.57E−06	4.04E−06
Cd	Child	8.05E−05	2.25E−07	4.44E−08
Cr	Child	4.50E−04	1.26E−06	2.48E−07
Cu	Child	1.15E−03	3.22E−06	6.33E−07
Fe	Child	1.66E−01	4.64E−04	9.14E−05
Ni	Child	2.52E−04	7.06E−07	1.39E−07
Pb	Child	6.19E−04	1.73E−06	3.41E−07
Zn	Child	3.66E−03	1.03E−05	2.02E−06

*Note: ADD* values recalculated using USEPA exposure factors (*BW* adults = 70 kg; children = 15 kg; *EF* = 350 days·year^−1^). Inhalation exposure is expressed as *EC* (mg·m^−3^), compatible with *RfC*_*i*_.

### 3.2 Noncarcinogenic Risk Characterization From Soil Exposure

The recalculated hazard quotients (*HQs*) indicated that adults generally had *HQ* values below unity across all exposure pathways (Table 2), indicating low noncarcinogenic risk under chronic exposure conditions. In contrast, children consistently presented higher *HQ* values, with soil ingestion being the primary contributor to risk, corroborating previous findings on age‐dependent vulnerability (Cabral‐Pinto et al. 2020; Ahmad et al. 2021).

**TABLE 2:** Hazard quotients (*HQs*) by exposure pathway and metal for children.

Metal	Ingestion *HQ*	Dermal *HQ*	Inhalation *HQ*
Cd	0.080	0.002	0.063
Cr	0.500	0.014	8.280
Cu	0.029	0.001	NA
Fe	0.237	0.001	NA
Ni	0.013	< 0.001	0.001
Pb	0.620	0.002	NA
Zn	0.012	< 0.001	NA

Abbreviation: NA: absence of *RfC*_*i*_ USEPA.

Iron (Fe) and lead (Pb) were the main contributors to cumulative risk in children. Although Fe is an essential element, its high concentrations in soil combined with conservative exposure assumptions resulted in elevated *HQ* values. Similar patterns have been reported in agricultural risk assessments, where essential metals disproportionately influence hazard indices due to high environmental abundance rather than intrinsic toxicity (Perković et al. 2022).

For children, soil ingestion was the dominant exposure pathway for all metals, whereas dermal contact contributed marginally to overall risk. Inhalation‐related *HQ* values exceeded unity only for chromium under a conservative USEPA *RfC*_*i*_ assumption, highlighting the sensitivity of this pathway to toxicological reference selection.

The *HI*, calculated as the sum of *HQ*s across all metals and exposure routes, exceeded unity for children in multiple sampling areas, indicating potential noncarcinogenic concern (Table 3). These results align with recent studies emphasizing cumulative exposure as a critical determinant of toxicological relevance in agricultural settings (S. Yang, Zhao, et al. 2019; Münzel et al. 2023).

**TABLE 3:** Hazard index (*HI*) from soil exposure in adults and children.

Area	Adult *HI*	Child *HI*
A1	0.245	1.950*
A2	0.134	1.006*
A3	0.107	0.857
A4	0.145	1.089*
A5	0.140	1.096*
A6	0.130	1.012*
A7	0.101	0.781
A8	0.108	0.878
A9	0.069	0.533
A10	0.073	0.570
A11	0.066	0.531
A12	0.098	0.773
A13	0.088	0.730
A14	0.116	0.953
A15	0.087	0.716
A16	0.108	0.870
A17	0.045	0.359
A18	0.098	0.739

*Note: HI* is the sum of pathway‐ and metal‐specific *HQ* values for soil exposure (ingestion + dermal + inhalation, with route‐specific reference values), recalculated using USEPA exposure factors (*BW* adults = 70 kg; children = 15 kg; *EF* = 350 days·year^‐1^). Values marked with * *HI > 1* indicate potential noncarcinogenic concern.

The spatial distribution of the HI highlights a clear contrast between adults and children, with no area exceeding the noncarcinogenic threshold for adults, whereas localized exceedances (HI > 1) were identified for children in five agricultural areas (Table 3). This pattern is consistent with the greater vulnerability of children and the dominance of soil ingestion as the primary exposure pathway. Although the present assessment was intentionally conservative and based on total metal concentrations, several studies have demonstrated that only a fraction of soil‐bound metals is bioaccessible under gastrointestinal conditions (Taati et al. 2020; Kumar et al. 2025; Lekuona‐Orkaizagirre et al. 2025). Conceptual sensitivity analysis indicates that applying realistic bioaccessibility fractions (e.g., 25%–50%) would substantially reduce HI values in marginal areas (e.g., A2 and A6), potentially shifting them below the threshold of concern, while the highest risk hotspot (A1) would likely remain above unity even under reduced bioaccessibility scenarios. These findings suggest that the identification of priority areas is robust, while also underscoring the role of bioaccessibility as a key source of uncertainty in HHRA. Consequently, the results should be interpreted as conservative upper‐bound estimates, suitable for screening‐level decision‐making and for guiding targeted soil management and public health interventions in smallholder farming systems.

### 3.3 Dietary Exposure Through Vegetable Consumption

Dietary intake via leafy vegetables represented a significant exposure route, particularly for Cd, Pb, and Cr.

Lettuce and arugula exhibited the highest CDI values, reflecting both elevated metal concentrations and higher CRs (Table 4). Comparable findings have been reported in studies assessing vegetable‐mediated exposure in peri‐urban farming systems (L. Yang, Liu, et al. 2019; Ahmad et al. 2021).

**TABLE 4:** Chronic daily intake (CDI, mg·kg^−1^·day^−1^) and hazard quotients (HQs) for vegetable consumption.

Crop	Metal	*CDI* (Adult)	*CDI* (Child)	*HQ* (Adult)	*HQ* (Child)
**Lettuce**	Cd	3.2 × 10^−4^	1.5 × 10^−3^	0.32	1.50*
	Pb	2.1 × 10^−3^	9.8 × 10^−3^	0.60	2.80*
	Cr	4.0 × 10^−4^	1.9 × 10^−3^	0.44	2.11*
**Arugula**	Cd	2.7 × 10^−4^	1.3 × 10^−3^	0.27	1.30*
	Pb	1.8 × 10^−3^	8.4 × 10^−3^	0.51	2.40*
	Cr	3.6 × 10^−4^	1.7 × 10^−3^	0.40	1.89*
**Mustard**	Cd	1.9 × 10^−4^	9.2 × 10^−4^	0.19	0.92
	Pb	1.2 × 10^−3^	5.6 × 10^−3^	0.34	1.60*
**Chives**	Cd	1.1 × 10^−4^	5.4 × 10^−4^	0.11	0.54
	Pb	9.5 × 10^−4^	4.4 × 10^−3^	0.27	1.26*

*Note: CDI* values were calculated using metal concentrations expressed on a fresh‐weight basis and age‐specific vegetable ingestion rates. Hazard quotients (*HQ*) were obtained by dividing *CDI* by the corresponding oral reference dose (*RfD*_ing_). Values marked with * *HQ* > 1 indicate potential noncarcinogenic concern.

Dietary exposure through leafy vegetable consumption contributed substantially to overall noncarcinogenic risk, particularly for children (Table 4). *CDI* values were consistently higher for children than for adults due to lower *BW* and higher intake rates relative to body mass. Lettuce and arugula exhibited the highest *CDI* values for cadmium (Cd), lead (Pb), and chromium (Cr), reflecting both elevated metal concentrations and frequent consumption patterns. While *HQ* values for adults remained below the threshold of concern (*HQ* < 1) for all crops and metals, several child‐specific *HQ* values exceeded unity, notably for Cd, Pb, and Cr in lettuce and arugula. These results are consistent with previous studies that have identified leafy vegetables as efficient vectors of metal exposure in peri‐urban and smallholder farming systems (Sultana et al. 2017; Atikpo et al. 2021; Osae et al. 2023). The findings underscore the heightened vulnerability of children to dietary metal exposure and reinforce the importance of incorporating age‐specific consumption patterns and fresh‐weight concentrations into HHRAs. Overall, dietary risk estimates should be interpreted as conservative upper‐bound values, given that metal bioaccessibility during digestion was not explicitly quantified.

### 3.4 Bioaccessibility Considerations and Conceptual Sensitivity Analysis

While total metal concentrations were used for conservative risk estimation, it is widely recognized that only a fraction of soil‐ and plant‐associated metals is bioaccessible following ingestion. Studies employing in vitro gastrointestinal simulations have demonstrated that bioaccessible fractions of Cd and Pb in soils often range between 10% and 40%, depending on soil pH, organic matter content, and metal speciation (Ruby et al. 1999; Mao et al. 2025).

In vegetables, bioaccessibility is influenced by plant matrix composition, cooking practices, and metal–ligand interactions. Studies have reported lower bioaccessibility for Cr and Fe relative to Cd and Pb, suggesting that risk estimates based on total concentrations likely overestimate actual internal exposure (Münzel et al. 2023).

To assess the sensitivity of risk estimates to bioaccessibility assumptions, a conceptual parametric analysis was conducted by applying hypothetical bioaccessibility factors (25%, 50%, and 75%) to the recalculated *ADD*/*CDI* values. This analysis demonstrated that, even under conservative reductions, *HQ* values for children remained above unity in several areas, indicating that the identification of potential health concerns is robust to reasonable variations in bioaccessibility assumptions.

Figure 2 illustrates the impact of assumed bioaccessibility fractions on the *HI*, showing how reductions in bioaccessible metal fractions modulate *HI* values while preserving the identification of child‐specific risk hotspots. The figure highlights a consistent adult–child contrast across all areas, with adult *HI* values remaining well below the screening threshold (*HI*  =  1) and child *HI* displaying pronounced spatial variability, including a clear hotspot at A1 (child *HI* ≈ 2.0) and marginal exceedances clustered around the decision boundary in areas A2, A4, A5, and A6. This pattern is characteristic of screening‐level HHRA applications and mirrors recent studies published, which report dominant child‐specific risks driven by ingestion pathways and localized contamination rather than area‐wide elevation (Addai‐Arhin et al. 2022). The sensitivity of near‐threshold areas (A2–A6) to uncertainty—particularly bioaccessibility and exposure behavior—supports a conservative, upper‐bound interpretation, whereas the persistence of the A1 hotspot across plausible scenarios reinforces its prioritization for risk management. Similar spatial contrasts and hotspot‐driven conclusions have been emphasized in recent assessments applying *HQ*/*HI* frameworks in environmental and occupational settings (Jarjees et al. 2025; Yasin and Salih 2025). Overall, the results support targeted mitigation focused on child‐sensitive hotspots while identifying marginal areas as candidates for refined, site‐specific assessment.

**FIGURE 2:**
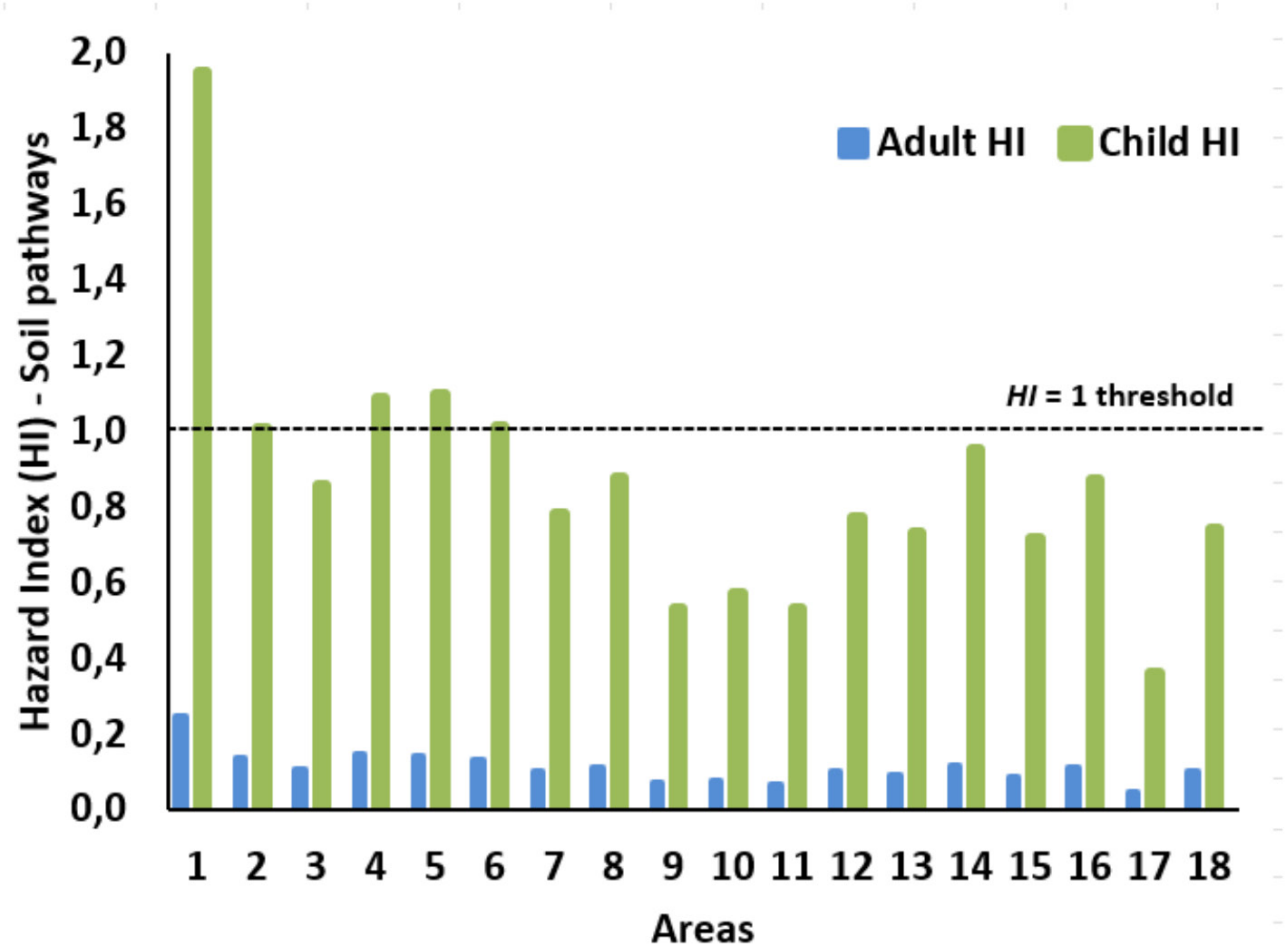
Conceptual sensitivity analysis of hazard index (*HI*) as a function of assumed bioaccessibility fractions.

### 3.5 Uncertainty, Limitations, and Toxicological Implications

The present assessment intentionally adopted conservative exposure assumptions to minimize the likelihood of underestimating health risks. Nevertheless, uncertainties remain, particularly regarding metal speciation, site‐specific bioaccessibility, and variability in dietary habits. As highlighted in recent discussions, such uncertainties are inherent to HHRA applications and should be interpreted as a basis for precaution rather than as definitive predictions of adverse outcomes (Tepanosyan et al. 2017; Perković et al. 2022).

Despite these limitations, the HHRA provides a methodologically robust and toxicologically defensible evaluation of human health risks in smallholder agricultural systems. The convergence of elevated *HI* values in children across multiple exposure pathways underscores the need for improved soil management, monitoring of agricultural inputs, and targeted public health interventions in peri‐urban farming regions.

The findings advance HHRA applications in environmental toxicology and provide transferable insights for risk management in similar agricultural systems worldwide.

## 4 Conclusions

This study applied a harmonized USEPA HHRA framework to evaluate noncarcinogenic exposure to PTMs in soils and leafy vegetables from smallholder agricultural systems in Northeastern Brazil. Soil ingestion and dietary consumption of leafy vegetables were identified as the dominant exposure pathways, with children consistently exhibiting higher *ADDs* and hazard indices than adults due to age‐specific exposure characteristics.

Noncarcinogenic risks for adults remained below the threshold of concern across all assessed areas, whereas localized exceedances of the *HI* were identified for children. These exceedances delineate spatially explicit risk hotspots rather than a uniform pattern of risk across the study area, reinforcing the importance of site‐specific and population‐specific risk evaluation. Lettuce and arugula emerged as the main contributors to dietary exposure, underscoring the role of crop type and consumption patterns in shaping overall risk.

The conceptual bioaccessibility sensitivity analysis demonstrated that risk estimates near regulatory thresholds are sensitive to realistic assumptions regarding bioaccessible metal fractions, whereas the highest risk hotspots remain robust under conservative exposure scenarios. Overall, the findings represent conservative, screening‐level estimates that highlight the relevance of age‐specific exposure pathways, spatially resolved risk assessment, and targeted management approaches in smallholder farming systems.

Future research should prioritize evaluating bioavailability‐informed risk models, assessing feasible soil and crop management strategies, and generating epidemiological evidence to refine long‐term health risk estimates. From a risk management perspective, the results support the need for targeted technical guidance to farmers and locally focused interventions that prioritize vulnerable populations, rather than uniform mitigation measures, in smallholder agricultural landscapes.

## Author Contributions

**Fernando Ferreira da Cunha Filho:** writing – original draft, methodology, investigation, formal analysis, conceptualization. **Alex Souza Moraes:** writing – review and editing, formal analysis. **João Paulo Siqueira da Silva:** methodology, investigation. **Edvane Borges da Silva:** writing – review and editing, visualization. **Ademir Amaral:** writing – review and editing, visualization. **André Maciel Netto:** writing – original draft, writing – review and editing, data curation, supervision, project administration, methodology, conceptualization, resources.

## Funding

The authors have nothing to report.

## Ethics Statement

This research did not involve human participants or animals and therefore did not require ethical approval.

## Conflicts of Interest

The authors declare no conflicts of interest.

## Supporting information




**Table S1:** Areas and geographic coordinates of collections in Vitória de Santo Antão—PE.
**Table S2:** Information on cultivated areas in Vitória de Santo Antão—PE.
**Table S3:** Description of fertilizers and pesticides used in the cultivation areas.
**Table S4:** Information on the crops collected and their respective areas.
**Table S5:** Chemical and physical characteristics of soil samples.
**Table S6:** Chemical properties, on a dry basis, of organic materials used in vegetable crops.
**Table S7:** Metal contents in soil samples collected in vegetable‐producing areas.
**Table S8:** Estimation of the increase of metals by agricultural inputs during 10 years of vegetable production.
**Table S9:** Metal contents in crop protection products are used in vegetable‐producing areas.
**Table S10:** Transfer factor (*t*) of soil metals to edible part of vegetable crops.
**Table S11:** Translocation index (*TI*) of metals from roots to edible parts of vegetables.
**Table S12:** Metal dose through inhalation of contaminated particles by adults and children.
**Table S13:** Metal dose through dermal contact with particles contaminated by adults and children.
**Table S14:** Available consumption rate (*CR*) of vegetables for adults and children.

## Data Availability

Data are available in the article supplementary material.
