# Supplementary material for: Human Health Risk Assessment From Exposure to Toxic Metals in Soils and Leafy Vegetables in Smallholder Farms of Northeastern Brazil
Source: J Appl Toxicol. 2026 Mar 19;46(11):3989–99. doi: 10.1002/jat.70164 (PMC13628439; doi:10.1002/jat.70164)
Supplement: Supplementary file 1 [file JAT-46-3989-s001.docx]

**Appendix A: Supplementary Material**

**Human Health Risk Assessment from Exposure to Toxic Metals in Soils and Leafy Vegetables in Smallholder Farms of Northeastern Brazil**

Fernando Ferreira da Cunha Filho^1^, Alex Souza Moraes^2^, João Paulo Siqueira da Silva^3^, Edvane Borges da Silva^4^, Ademir Amaral^5^, André Maciel Netto^5*^

^1^Federal Institute of Education, Science and Technology of Pernambuco, Campus Belo Jardim, Pernambuco, Brazil;

^2^Department of Chemistry, Federal Rural University of Pernambuco, Recife, Pernambuco, Brazil;

^3^Pernambuco Department of Education, Recife, Pernambuco, Brazil;

^4^Academic Center of Vitória, Federal University of Pernambuco, Vitoria de Santo Antão, Pernambuco, Brazil;

^5^Department of Nuclear Energy, Federal University of Pernambuco, Recife, Pernambuco, Brazil

**Corresponding author:**

André Maciel Netto

Department of Nuclear Energy, Federal University of Pernambuco, Av. Prof. Luiz Freire, 1000 – Curado, PE, 50740-545, Brazil; andre.netto@ufpe.br or andmnetto@gmail.com

ORCID: 0000-0002-1341-5171

This Supplementary Material supports and complements the accompanying manuscript entitled “***Human Health Risk Assessment from Exposure to Toxic Metals in Soils and Leafy Vegetables in Smallholder Farms of Northeastern Brazil***” by providing detailed methodological descriptions, site characterization, analytical procedures, exposure parameters, and supporting datasets used in the human health risk assessment (HHRA), and is intended solely to document how data were generated and processed without presenting or interpreting results.

**Study area, sampling design, and analytical procedures**

The Supplementary Material details the study area, sampling locations, and sample collection strategy for soils and leafy vegetables collected from 18 smallholder agricultural areas in Vitória de Santo Antão (PE, Brazil).

**Table S1.** Areas and geographic coordinates of collections in Vitória de Santo Antão – PE.

| **Area** | **Acronym** | **Coordinates** |
| --- | --- | --- |
| Natuba (near the Dump) | A1 | 8°08'44.6"S 35°18'42.5"W |
| Natuba – próximo a BR 232 | A2 | 8°08'36.3"S 35°18'27.8"W |
| Natuba | A3 | 8°08'15.6"S 35°18'52.6"W |
| Natuba – Tapacurá River | A4 | 8°08'03.9"S 35°18'11.0"W |
| Cedro | A5 | 8°07'51.5"S 35°18'04.6"W |
| Ladeira de Pedra | A6 | 8°08'13.7"S 35°16'59.4"W |
| Galileia | A7 | 8°08'00.2"S 35°15'39.0"W |
| Engenho Genipapo | A8 | 8°10'09.2"S 35°15'47.9"W |
| Laranjeirinha | A9 | 8°10'10.4"S 35°18'06.8"W |
| Mocotó | A10 | 8°11'41.2"S 35°19'40.4"W |
| Mocotó | A11 | 8°11'14.0"S 35°20'12.3"W |
| Engenho Pitú | A12 | 8°12'21.6"S 35°20'25.0"W |
| Serra Grande | A13 | 8°12'04.6"S 35°20'54.1"W |
| Cacimbas | A14 | 8°03'56.0"S 35°16'00.3"W |
| Oiteiro | A15 | 8°03'58.4"S 35°18'24.5"W |
| Oiteiro | A16 | 8°04'10.9"S 35°17'42.2"W |
| Chã de Serraria | A17 | 8°04'48.0"S 35°19'45.7"W |
| Pirituba | A18 | 8°04'22.1"S 35°21'16.6"W |

**Table S2.** Information on cultivated areas in Vitória de Santo Antão -PE

| **Areas** | **Cultivated area (ha)** | **Crops** | **Cultivation time (years)** |
| --- | --- | --- | --- |
| A1 | 2.5 | Lettuce, coriander, chives, arugula, cucumber, radish, bredo, mustard, mint and pepper. | 26 |
| A2 | 2 | Lettuce, coriander, chives, arugula, cucumber, radish, bredo, mint, mustard and pepper. | 31 |
| A3 | 5.5 | Lettuce, coriander, chives, arugula, cucumber, radish, bredo, mint and pepper. | 21 |
| A4 | 3.5 | Lettuce, coriander, chives, arugula, cucumber, mustard, radish, bredo, mint and pepper. | 22 |
| A5 | 2.5 | Lettuce, coriander, chives, arugula, mint and pepper. | 16 |
| A6 | 5 | Lettuce, cilantro, chives, and arugula. | 21 |
| A7 | 6 | Lettuce, cilantro, chives, arugula and chard | 26 |
| A8 | 2 | Lettuce, coriander, chives, arugula, cucumber, turnip. | 31 |
| A9 | 3.5 | Lettuce, cilantro, chives. | 31 |
| A10 | 4 | Lettuce, coriander, chives, arugula and chard. | 21 |
| A11 | 6.5 | Lettuce, cilantro, chives, arugula, cucumber. | 18 |
| A12 | 4.5 | Lettuce, cilantro, chives, tomatoes and okra | 26 |
| A13 | 3.5 | Lettuce, coriander, chives, mint and chard. | 31 |
| A14 | 2.5 | Lettuce, coriander, chives, arugula, chard and mustard | 28 |
| A15 | 4 | Lettuce, coriander, chives, chard and pepper. | 24 |
| A16 | 4.5 | Lettuce, cilantro, chives, arugula, cucumber and tomato. | 28 |
| A17 | 5 | Lettuce, chives, arugula, mint and zucchini | 18 |
| A18 | 3.5 | Lettuce, coriander, arugula, mint and zucchini | 31 |

Table S3. Description of fertilizers and pesticides used in the cultivation areas.

| Product | Class and composition | Dose (ha) | Frequency of applications |
| --- | --- | --- | --- |
| NPK 15-11-11 | Fertilizer - N 15%, P 11% e K11% | 50 kg | 1 |
| Fertilizer 1 | Fertilizer – Cu 20%, S 10% e Ca 3% | 0.3 kg | 4 |
| Fertilizer 2 | Fertilizer - N 6% e P 30% | 2 L | 2 |
| Fertilizer 3 | Fertilizer - 15% P, 8% K, Zn 2%, Cu 0,3% | 3 L | 1 |
| Fertilizer 4 | Fertilizer - P 20%, Cu 3,5% e S 1,75% | 2 L | 1 |
| Fertilizer 5 | Fertilizer - NPK 20% e Fe + B + Zn 1% | 2 L | 1 |
| Insecticide 1 | Insecticide - Imidacloprid | 0.3 kg | 4 |
| Insecticide 2 | Insecticide and acaricide - ABAMECTIN | 0.1 L | 5 |
| Herbicide | Systemic Herbicide - FLUAZIFOPE-P-BUTYL | 0.5 L | 3 |
| Fungicide 1 | Microbiological bactericidal fungicide - Bacillus subtilis | 2 L | 2 |
| Fungicide 2 | Systemic Fungicide - AZOXYSTROBIN | 0.12 kg | 4 |
| Bovine manure | Organic fertilizer | 800 kg | 1 |
| Chicken litter | Organic fertilizer | 500 kg | 1 |

Table S4. Information on the crops collected and their respective areas.

Continua

| Culture | Scientific Name | Production | Organ analyzed | Collection area |
| --- | --- | --- | --- | --- |
| Victoria Lettuce (smooth) | *Lactuca sativa L.* | 20000 pls ha^-1^ | Root, stem and leaves (edible part) | A1, A2 e A4 |
| Victoria Lettuce (smooth) | *Lactuca sativa L.* | 20000 pls ha^-1^ | Leaves (edible part) | A1 a A18 |
| Mimosa Lettuce (purple) | *Lactuca sativa L.* | 15000 pls ha^-1^ | Root, stem, and leaves (edible part) | A1, A2 e A4 |
| Cristina Lettuce (curly) | *Lactuca sativa L.* | 17000 pls ha^-1^ | Root, stem, and leaves (edible part) | A1, A2 e A4 |
| Lettuce (American) Great Lakes | *Lactuca sativa L.* | 6000 pls ha^-1^ | Root, stem, and leaves (edible part) | A1, A2 e A4 |
| Cucumber Caipira | *Cucumis sativus L.* | 20 t ha^-1^ | Root, stem, leaves, flower and fruit (edible part) | A1, A2 e A4 |
| Cucumber Aodai | *Cucumis sativus L.* | 18 t ha^-1^ | Root, stem, leaves, flower and fruit (edible part) | A1, A2 e A4 |
| Chives Every Year | *Allium Schoenoprasum L.* | 15000 bunch ha^-1^ | Root and edible part (bulb and leaf, separately) | A1, A2 e A4 |
| Rabanete Giant Crimson | *Raphanus sativus* | 10000 bunch ha^-1^ | Root (edible part), Stem, Leaf | A1, A2 e A4 |
| Mustard | Brassica juncea (L.) Coss. | 20000 bunch ha^-1^ | Root, Stem, Leaf (Edible Part) and Flower | A1, A2 e A4 |
| Cultivated Arugula | *Eruca sativa L.* | 1000 bunch ha^-1^ | Root and Leaf (Edible Part) | A1, A2 e A4 |

Descriptions include geographic coordinates, cultivated crops, and agronomic practices, without interpretation of spatial trends or health relevance.

**Soil and vegetable chemical characterization**

Soil physicochemical properties (pH, organic matter, cation exchange capacity, particle size distribution) and metal concentrations are reported as analytical input data for HHRA calculations.

**Table S5.** Chemical and physical characteristics of soil samples.

| **Areas** | **pH**  **(H_2_O)** | **Ca^2+^** | **Mg^2+^** | | **K^+^** | **Na^+^** | **SB** | **Al^3+^** | **CTC_e_** | **(H+Al)** | **CTC pH 7,0** | **V** | **m** | **COT** | **MO** | **P** | **Sand** | **Loam** | **Clay** |
| --- | --- | --- | --- | --- | --- | --- | --- | --- | --- | --- | --- | --- | --- | --- | --- | --- | --- | --- | --- |
|  |  | **-------------------------------------cmol_c_ dm^3^------------------------------------------** | | | | | | | | | | **--------%--------** | | **-------g kg^-1^-----** | | **mg dm^3^** | **------------g kg^-1^-----------** | | |
| A1 | 6.77 | 7.90 | | 5.67 | 0.16 | 1.44 | 15.16 | 0.18 | 15.35 | 4.01 | 19.17 | 79.10 | 1.19 | 29.42 | 50.71 | 1281.46 | 564.08 | 306.96 | 128.95 |
| A2 | 7.15 | 7.67 | | 5.37 | 1.84 | 0.83 | 15.70 | 0.33 | 16.04 | 2.97 | 18.67 | 84.09 | 2.08 | 20.10 | 34.65 | 785.34 | 547.19 | 347.29 | 105.52 |
| A3 | 7.11 | 6.17 | | 6.03 | 1.04 | 0.88 | 14.11 | 0.26 | 14.38 | 2.04 | 16.15 | 87.41 | 1.84 | 19.16 | 33.03 | 782.18 | 576.74 | 341.19 | 82.07 |
| A4 | 7.12 | 10.00 | | 4.22 | 1.42 | 0.85 | 16.48 | 0.30 | 16.78 | 2.56 | 19.04 | 86.58 | 1.79 | 17.81 | 30.70 | 703.47 | 601.24 | 302.76 | 96.00 |
| A5 | 6.23 | 12.40 | | 5.02 | 1.89 | 0.78 | 20.09 | 0.15 | 20.24 | 3.03 | 23.12 | 86.89 | 0.74 | 20.94 | 36.11 | 964.17 | 617.03 | 288.41 | 94.56 |
| A6 | 6.92 | 9.90 | | 5.02 | 1.16 | 0.91 | 16.99 | 0.10 | 17.09 | 4.40 | 21.39 | 79.43 | 0.59 | 16.90 | 29.14 | 873.45 | 598.36 | 274.69 | 126.95 |
| A7 | 7.25 | 8.30 | | 4.51 | 1.18 | 0.31 | 14.30 | 0.10 | 14.41 | 3.63 | 17.93 | 79.77 | 0.72 | 16.06 | 27.69 | 927.38 | 515.05 | 355.99 | 128.96 |
| A8 | 6.18 | 6.57 | | 4.25 | 0.73 | 0.11 | 11.65 | 0.20 | 11.85 | 3.63 | 15.29 | 76.24 | 1.69 | 13.56 | 23.37 | 843.79 | 451.00 | 454.13 | 94.87 |
| A9 | 7.00 | 8.50 | | 5.43 | 0.86 | 0.13 | 14.92 | 0.20 | 15.12 | 2.90 | 17.81 | 83.74 | 1.32 | 5.92 | 10.20 | 607.88 | 649.97 | 221.07 | 128.96 |
| A10 | 6.68 | 8.33 | | 4.47 | 0.76 | 0.17 | 13.73 | 0.10 | 13.83 | 3.41 | 17.14 | 80.11 | 0.72 | 12.04 | 20.75 | 761.28 | 545.00 | 361.93 | 93.07 |
| A11 | 6.51 | 11.35 | | 9.00 | 1.42 | 0.54 | 22.31 | 0.10 | 22.41 | 4.22 | 26.53 | 84.11 | 0.45 | 18.24 | 31.44 | 899.37 | 486.00 | 376.00 | 138.00 |
| A12 | 5.55 | 4.78 | | 5.22 | 0.25 | 0.03 | 10.28 | 0.10 | 10.38 | 4.46 | 14.74 | 69.77 | 0.96 | 22.59 | 38.94 | 979.28 | 553.40 | 341.50 | 105.10 |
| A13 | 6.62 | 11.47 | | 6.24 | 2.66 | 0.60 | 20.97 | 0.20 | 21.17 | 4.26 | 25.23 | 83.11 | 0.94 | 12.55 | 21.64 | 891.35 | 511.60 | 328.00 | 160.40 |
| A14 | 6.97 | 9.83 | | 4.76 | 1.26 | 0.60 | 16.45 | 0.00 | 16.45 | 3.47 | 19.91 | 82.60 | 0.00 | 24.64 | 42.49 | 769.29 | 613.10 | 297.00 | 89.90 |
| A15 | 6.38 | 6.90 | | 2.84 | 1.12 | 0.24 | 16.48 | 0.00 | 11.10 | 3.08 | 14.18 | 78.29 | 0.00 | 7.82 | 13.48 | 336.35 | 494.50 | 371.60 | 133.90 |
| A16 | 6.09 | 3.30 | | 1.0 | 0.61 | 0.14 | 5.05 | 0.10 | 5.15 | 3.04 | 8.09 | 62.42 | 1.94 | 19.84 | 34.21 | 108.60 | 579.00 | 327.50 | 93.50 |
| A17 | 5.93 | 6.93 | | 3.43 | 0.48 | 0.10 | 10.94 | 0.10 | 11.04 | 5.52 | 16.45 | 66.49 | 0.91 | 16.38 | 28.23 | 156.01 | 632.40 | 284.00 | 83.60 |
| A18 | 5.65 | 7.35 | | 1.41 | 0.46 | 0.20 | 9.42 | 0.10 | 9.52 | 6.88 | 16.30 | 57.80 | 1.05 | 12.57 | 21.67 | 281.66 | 581.40 | 338.20 | 80.40 |
| Minimum | 5.55 | 3.30 | | 1.0 | 0.16 | 0.03 | 5.05 | 0.00 | 5.15 | 2.04 | 8.09 | 57.80 | 0.00 | 5.92 | 10.20 | 108.60 | 451.00 | 221.07 | 80.40 |
| Maximum | 7.25 | 12.40 | | 9.0 | 2.66 | 1.44 | 22.31 | 0.33 | 22.41 | 6.88 | 26.53 | 87.41 | 2.08 | 29.42 | 50.71 | 1281.46 | 649.97 | 454.13 | 160.40 |
| Mean | 6.56 | 8.20 | | 4.61 | 1.05 | 0.49 | 14.35 | 0.18 | 14.53 | 3.75 | 18.09 | 77.77 | 1.28 | 17.03 | 29.36 | 719.57 | 562.06 | 328.79 | 109.15 |
| Standard deviation | 0.53 | 2.31 | | 1.88 | 0.65 | 0.39 | 4.42 | 0.13 | 4.48 | 1.12 | 4.39 | 9.48 | 0.81 | 5.67 | 9.78 | 304.85 | 54.27 | 50.11 | 23.35 |

**Table S6.** Chemical properties, on a dry basis, of organic materials used in vegetable crops.

| **Attributes** | **Cattle Manure** | **Chicken**  **litter** |
| --- | --- | --- |
| **g kg^-1^** | | |
| OM | 596.1 | 854.41 |
| OC | 300 | 430 |
| N | 13.9 | 23.41 |
| K | 13.6 | 25.34 |
| P | 13.5 | 16.28 |
| Ca | 16.9 | 20.14 |
| Mg | 4.9 | 6.87 |
| Fe | 3.2 | 5.66 |
|  | mg kg^-1^ | |
| Zn | 149.00 | 265.78 |
| Cu | 30.00 | 146.82 |
| Mn | 458.70 | 352.49 |
| B | 16.10 | 22.15 |
| Cd | 0.83 | 6.00 |
| Pb | 18.21 | 34.00 |
| Cr | 16.50 | 27.98 |
| Ni | 43.00 | 52.87 |
| Relation C/N | 22/1 | 13/1 |
| pH CaCl_2_ | 7.2 | 8.15 |

Vegetable samples were analyzed using the same digestion and instrumental procedures as soils, and metal concentrations are provided solely as exposure inputs.

**Quality assurance and quality control**

Analytical quality assurance and quality control (QA/QC) procedures are documented, including the use of certified reference materials, analytical blanks, and duplicate samples.

**Table S7.** Metal contents in soil samples collected in vegetable producing areas.

| **Areas** | **Cd** | **Cr** | **Cu** | **Fe** | **Ni** | **Pb** | **Zn** | |
| --- | --- | --- | --- | --- | --- | --- | --- | --- |
|  | **mg kg ^-1^** | | | | | | |  |
| A1 | 4.50 | 58.64 | 181.64 | 36558.60 | 34.52 | 82.44 | 567.62 | |
| A2 | 2.50 | 41.61 | 139.62 | 9860.07 | 26.32 | 33.01 | 462.64 | |
| A3 | 1.50 | 26.78 | 107.38 | 16120.00 | 11.41 | 30.44 | 332.28 | |
| A4 | 3.50 | 43.25 | 50.75 | 10537.11 | 33.56 | 54.53 | 185.12 | |
| A5 | 0.81 | 40.20 | 103.48 | 18403.67 | 17.98 | 31.95 | 386.88 | |
| A6 | 3.01 | 36.48 | 116.22 | 13433.33 | 18.24 | 40.53 | 419.12 | |
| A7 | 0.58 | 29.66 | 98.28 | 10934.73 | 14.29 | 26.98 | 330.98 | |
| A8 | 0.34 | 27.66 | 125.32 | 17033.47 | 5.23 | 30.50 | 453.78 | |
| A9 | 0.28 | 17.91 | 59.36 | 8430.76 | 14.83 | 23.47 | 221.52 | |
| A10 | 0.13 | 19.67 | 52.34 | 8371.65 | 11.53 | 30.94 | 110.50 | |
| A11 | 0.09 | 15.51 | 107.64 | 8288.37 | 8.88 | 31.06 | 281.06 | |
| A12 | 0.07 | 22.02 | 123.50 | 13863.20 | 21.25 | 38.77 | 436.05 | |
| A13 | 0.71 | 17.02 | 74.41 | 12205.53 | 7.87 | 65.58 | 243.88 | |
| A14 | 0.18 | 24.16 | 136.76 | 17817.97 | 13.08 | 62.51 | 467.48 | |
| A15 | 0.12 | 16.94 | 53.46 | 12337.17 | 11.89 | 64.66 | 211.64 | |
| A16 | 0.16 | 22.60 | 54.05 | 14618.15 | 16.64 | 73.94 | 124.28 | |
| A17 | 0.02 | 9.72 | 31.95 | 8060.00 | 6.76 | 16.22 | 57.24 | |
| A18 | 0.13 | 33.82 | 65.96 | 2269.74 | 14.06 | 51.07 | 249.34 | |
| Minimum | 0.02 | 9.72 | 31.95 | 2269.74 | 5.23 | 16.22 | 57.24 | |
| Maximum | 4.52 | 58.64 | 181.64 | 36558.60 | 34.52 | 82.44 | 567.62 | |
| Average | 1.02 | 27.98 | 93.45 | 13285.69 | 16.02 | 43.81 | 307.85 | |
| SD | 1.36 | 12.13 | 39.43 | 6980.12 | 8.20 | 18.83 | 140.73 | |
| CV (%) | 133.33 | 43.35 | 42.19 | 52.54 | 51.18 | 42.98 | 45.71 | |
| QRV | 0.5 | 35.0 | 5.0 | - | 9.0 | 13.0 | 35.0 | |
| PV | 1.3 | 75.0 | 60.0 | - | 30.0 | 72.0 | 300.0 | |
| IV | 3.0 | 150.0 | 200.0 | - | 70.0 | 180.0 | 450.0 | |

SD: Standard deviation; CV: Coefficient of variation; QRV: Quality Reference Value for soils in Pernambuco (CPRH, 2014); PV: Prevention Value (CONAMA, 2009); IV: Research Value for the agricultural scenario (CONAMA, 2009); - Unspecified values.

**Table S8.** Estimation of the increase of metals by agricultural inputs during ten years of vegetable production.

| **Inputs** | **Cd** | **Cr** | **Cu** | **Fe** | **Ni** | **Pb** | **Zn** |
| --- | --- | --- | --- | --- | --- | --- | --- |
|  | **g ha^-1^** | | | | | | |
| NPK 15-11-11 | 1.33E+01 | 9.79E+01 | 1.20E-02 | 4.27E+01 | 4.10E+01 | 2.58E+00 | 3.60E-02 |
| Fertilizer 1 | 3.17E-02 | 3.73E-01 | 8.83E-02 | 3.93E-02 | 6.12E-01 | 1.18E-01 | 1.30E-03 |
| Fertilizer 2 | 2.01E+00 | 1.01E+02 | 3.14E-01 | 2.94E+01 | 1.79E+02 | 1.49E-01 | 1.34E-02 |
| Fertilizer 3 | 1.39E+01 | 7.38E-01 | 6.48E-01 | 7.99E-02 | 9.10E+00 | 4.43E-01 | 7.13E-02 |
| Fertilizer 4 | 2.64E-02 | 1.10E+01 | 2.81E+00 | 2.14E-01 | 5.12E+00 | 5.38E-01 | 4.80E-04 |
| Fertilizer 5 | 9.60E-03 | 2.78E-01 | 2.26E-02 | 5.18E-02 | 4.71E+01 | 2.40E-03 | 1.97E+00 |
| Insecticide 1 | 2.88E-03 | 6.05E-02 | 8.99E-01 | 1.68E+01 | 5.47E-02 | 9.79E-02 | 9.98E-01 |
| Insecticide 2 | 3.60E-03 | 1.20E-03 | 1.16E+01 | 2.60E+01 | 2.54E-01 | 1.50E-02 | 7.20E-02 |
| Herbicide | 0.00E+00 | 8.14E-01 | 5.12E+00 | 8.22E+02 | 3.60E-02 | 1.66E-01 | 7.59E+00 |
| Fungicide 1 | 4.80E-03 | 9.60E-03 | 1.00E+02 | 2.71E+02 | 3.41E-01 | 1.44E-02 | 4.80E-02 |
| Fungicide 2 | 0.00E+00 | 2.49E-01 | 5.41E-01 | 9.42E+01 | 6.16E-01 | 2.52E-01 | 5.77E-01 |
| Cattle Manure | 7.97E+01 | 1.58E+03 | 2.88E+03 | 3.07E+05 | 4.13E+03 | 1.75E+03 | 1.43E+04 |
| Chicken litter | 3.60E+02 | 1.68E+03 | 8.81E+03 | 3.40E+05 | 3.17E+03 | 2.04E+03 | 1.59E+04 |

**Table S9.** Metal contents in crop protection products are used in vegetable producing areas.

| **Agricultural Defensive** | **Cd** | **Cr** | **Cu** | **Fe** | **Ni** | **Pb** | **Zn** |
| --- | --- | --- | --- | --- | --- | --- | --- |
|  | **mg kg^-1^** | | | | | | |
| Inseticide 1 | 0.02 | 0.42 | 6.24 | 116.33 | 0.38 | 0.68 | 6.93 |
| Inseticide 2 | 0.06 | 0.02 | 193.45 | 433.51 | 4.23 | 0.25 | 1.20 |
| Herbicide | 0.00 | 4.52 | 28.47 | 4565.33 | 0.20 | 0.92 | 42.16 |
| Fungicide 1 | 0.01 | 0.02 | 209.04 | 564.61 | 0.71 | 0.03 | 0.10 |
| Fungicide 2 | 0.00 | 4.32 | 9.40 | 1636.18 | 10.70 | 4.37 | 10.02 |

Metal recoveries and analytical precision are reported to demonstrate data reliability.

**Exposure parameters and toxicological reference values**

All exposure parameters and toxicological reference values used in the HHRA are compiled in supplementary tables and were selected exclusively from USEPA guidance documents (RAGS, Exposure Factors Handbook, IRIS).

**Table S10.** Transfer Factor (*t*) of soil metals to edible part of vegetable crops**.**

| **Culture** | **Statistics** | **Cd** | **Cr** | **Cu** | **Fe** | **Ni** | **Pb** | **Zn** |
| --- | --- | --- | --- | --- | --- | --- | --- | --- |
| Arugula | Mean | 0.52±0.14 | 0.10±0.03 | 0.09±0.03 | 0.01±0.0 | 0.02±0.01 | 0.05±0.02 | 0.13±0.04 |
|  | Range | 0.42±0.68 | 0.06±0.13 | 0.04±0.11 | 0.01±0.02 | 0.01±0.03 | 0.04±0.07 | 0.08±0.18 |
| Iceberg lettuce | Mean | 0.15±0.16 | 0.14±0.04 | 0.14±0.08 | 0.02±0.01 | 0.16±0.12 | 0.07±0.03 | 0.08±0.03 |
|  | Range | 0.01±0.34 | 0.10±0.19 | 0.04±0.20 | 0.01±0.02 | 0.04±0.28 | 0.04±0.11 | 0.04±0.10 |
| Purple lettuce | Mean | 0.07±0.02 | 0.09±0.05 | 0.140.017 | 0.01±0.01 | 0.13±0.11 | 0.04±0.01 | 0.08±0.01 |
|  | Range | 0.04±0.10 | 0.03±0.12 | 0.12±0.16 | 0.00±0.02 | 0.02±0.24 | 0.02±0.05 | 0.07±0.10 |
| Smooth lettuce | Mean | 0.15±0.12 | 0.15±0.04 | 0.13±0.07 | 0.01±0.005 | 0.14±0.13 | 0.04±0.01 | 0.09±0.03 |
|  | Range | 0.01±0.25 | 0.12±0.20 | 0.04±0.19 | 0.01±0.02 | 0.01±0.27 | 0.02±0.06 | 0.05±0.12 |
| Curly lettuce | Mean | 0.09±0.11 | 0.18±0.09 | 0.28±0.05 | 0.01±0.001 | 0.12±0.09 | 0.04±0.01 | 0.10±0.038 |
|  | Range | 0.02±0.23 | 0.28±0.28 | 0.34±0.34 | 0.01±0.01 | 0.20­±0.20 | 0.06±0.06 | 0.15±0.15 |
| Chives | Mean | 0.08±0.09 | 0.15±0.035 | 0.21±0.06 | 0.01±0.01 | 0.03±0.02 | 0.02±0.0 | 0.18±0.051 |
|  | Range | 0.03±0.19 | 0.12±0.19 | 0.14±0.27 | 0.01±0.02 | 0.02±0.06 | 0.01±0.02 | 0.15±0.24 |
| Radish | Mean | 0.02±0.01 | 0.11±0.07 | 0.26±0.04 | 0.01±0.01 | 0.02±0.01 | 0.02±0.01 | 0.14±0.05 |
|  | Range | 0.01±0.03 | 0.03±0.18 | 0.23±0.31 | 0.01±0.02 | 0.01±0.03 | 0.01±0.03 | 0.08±0.20 |
| Aodai Cucumber | Mean | 0.10±0.07 | 0.18±0.06 | 0.14±0.05 | 0.01±0.0 | 0.06±0.02 | 0.02±0.01 | 0.14±0.05 |
|  | Range | 0.04±0.19 | 0.11±0.24 | 0.08±0.19 | 0.01±0.01 | 0.03±0.09 | 0.01±0.02 | 0.08±0.19 |
| Country Cucumber | Mean | 0.05±0.02 | 0.03±0.02 | 0.13±0.06 | 0.01±0.01 | 0.15±0.11 | 0.02±0.01 | 0.10±0.04 |
|  | Range | 0.01±0.07 | 0.01±0.06 | 0.06±0.20 | 0.00±0.03 | 0.04±0.27 | 0.01±0.03 | 0.05±0.13 |
| Mustard | Mean | 0.17±0.13 | 0.26±0.07 | 0.45±0.09 | 0.06±0.03 | 0.10±0.04 | 0.03±0.01 | 0.28±0.06 |
|  | Range | 0.05±0.31 | 0.17±0.31 | 0.34±0.52 | 0.03±0.09 | 0.06±0.14 | 0.02±0.03 | 0.24±0.35 |

Edible part – Arugula: leaf; Lettuce: leaf; Chives: leaf and stem; Radish: root; Cucumber: fruit; Mustard: stem, leaf and flower.

**Table S11.** Translocation Index (*TI*) of metals from roots to edible parts of vegetables.

| **Culture** | **Statistics** | **Cd** | **Cr** | **Cu** | **Fe** | **Ni** | **Pb** | **Zn** |
| --- | --- | --- | --- | --- | --- | --- | --- | --- |
| Arugula | Mean | 13.16±2.85 | 2.13±0.44 | 0.22±0.01 | 0.96±0.23 | 0.44±0.17 | 1.56±0.19 | 0.75±0.13 |
|  | Range | 11.20±16.43 | 1.84±2.64 | 0.21±0.23 | 0.74±1.20 | 0.33±0.65 | 1.41±1.78 | 0.59±0.84 |
| Iceberg lettuce | Mean | 10.59±10.44 | 0.55±0.16 | 1.03±0.11 | 0.89±0.56 | 1.24±0.69 | 3.72±0.29 | 1.93±0.44 |
|  | Range | 1.44±24.76 | 0.40±0.72 | 0.96±1.16 | 0.27±1.37 | 0.47±1.82 | 3.39±3.94 | 1.58±2.43 |
| Purple lettuce | Mean | 2.65±1.44 | 0.72±0.28 | 1.24±0.23 | 3.33±1.12 | 2.32±1.58 | 2.04±1.01 | 1.37±0.13 |
|  | Range | 1.50±4.27 | 0.39±0.89 | 1.04±1.50 | 2.54±4.60 | 0.73±3.89 | 0.93±2.89 | 1.26±1.52 |
| Smooth lettuce | Mean | 7.75±10.01 | 2.10±1.047 | 1.41±0.75 | 2.20±0.848 | 2.48±1.48 | 2.84±0.55 | 1.45±0.08 |
|  | Range | 1.46±19.29 | 0.97±3.04 | 0.90±2.28 | 1.65±3.17 | 1.04±4.01 | 2.26±3.37 | 1.35±1.51 |
| Curly Lettuce | Mean | 2.64±2.22 | 0.53±0.12 | 2.00±0.82 | 0.89±0.40 | 2.25±1.60 | 1.70±0.78 | 2.11±0.70 |
|  | Range | 1.03±5.17 | 0.39±0.62 | 1.35±2.93 | 0.57±1.34 | 0.81±3.97 | 0.80±2.22 | 1.55±2.91 |
| Chives | Mean | 7.96±11.45 | 1.48±1.046 | 0.59±0.09 | 0.83±0.216 | 0.52±0.11 | 1.24±0.76 | 2.06±0.19 |
|  | Range | 0.80±21.17 | 0.81±2.68 | 0.49±0.68 | 0.64±1.06 | 0.43±0.65 | 0.50±2.02 | 1.84±2.18 |
| Radish | Mean | 3.19±2.47 | 1.31±0.42 | 0.69±0.31 | 3.05±1.17 | 3.05±0.86 | 2.25±1.10 | 1.49±0.17 |
|  | Range | 1.32±6.00 | 1.01±1.80 | 0.35±0.97 | 2.14±4.38 | 2.28±3.98 | 1.01±3.13 | 1.38±1.70 |
| Cucumber Aodai | Mean | 2.54±2.80 | 0.94±0.06 | 3.59±0.59 | 10.74±0.88 | 3.69±1.89 | 2.43±0.51 | 3.43±0.48 |
|  | Range | 0.90±5.78 | 0.86±0.98 | 3.17±4.27 | 9.76±11.46 | 2.60±5.87 | 1.93±2.95 | 2.89±3.82 |
| Country cucumber | Mean | 5.21±2.63 | 1.57±0.58 | 2.78±1.05 | 3.32±1.18 | 5.12±1.86 | 2.81±0.81 | 3.77±1.04 |
|  | Range | 3.59±8.25 | 1.00±2.17 | 1.80±3.90 | 2.30±4.62 | 3.23±6.95 | 2.07±3.68 | 3.01±4.96 |
| Mustard | Mean | 9.71±5.85 | 14.54±1.69 | 5.58±1.86 | 10.24±1.94 | 5.37±0.32 | 2.44±0.42 | 3.67±1.21 |
|  | Range | 3.95±15.48 | 13.05±16.00 | 3.72±7.43 | 7.68±11.50 | 4.84±5.47 | 2.26±3.10 | 2.48±4.67 |

This section serves only to document parameter selection and does not interpret risk outcomes.

**Supporting HHRA calculations**

Table S12. Metal dose through inhalation of contaminated particles by adults and children.

| **Target audience** | **Statistic** | **Cd** | **Cr** | **Cu** | **Fe** | **Ni** | **Pb** | **Zn** |
| --- | --- | --- | --- | --- | --- | --- | --- | --- |
|  |  | **μg kg^-1^ dia^-1^** | | | | | | |
| **ADULT** | **Mean** | 4.34E-07 | 1.17E-05 | 3.92E-05 | 5.58E-03 | 6.72E-06 | 1.84E-05 | 1.29E-04 |
|  | **Minimum** | 8.39E-09 | 4.08E-06 | 1.34E-05 | 9.53E-04 | 2.19E-06 | 6.81E-06 | 2.40E-05 |
|  | **Maximum** | 1.89E-06 | 2.46E-05 | 7.62E-05 | 1.53E-02 | 1.45E-05 | 3.46E-05 | 2.38E-04 |
|  | **Standard deviation** | 5.81E-07 | 5.19E-06 | 1.69E-05 | 2.99E-03 | 3.51E-06 | 8.05E-06 | 6.02E-05 |
|  | **CV (%)** | 132.95 | 44.36 | 43.11 | 53.58 | 52.23 | 43.75 | 46.67 |
| **CHILD** | **Mean** | 3.04E-07 | 8.22E-06 | 2.75E-05 | 3.90E-03 | 4.71E-06 | 1.29E-05 | 9.04E-05 |
|  | **Minimum** | 5.88E-09 | 2.86E-06 | 9.39E-06 | 6.67E-04 | 1.54E-06 | 4.77E-06 | 1.68E-05 |
|  | **Maximum** | 1.32E-06 | 1.72E-05 | 5.34E-05 | 1.07E-02 | 1.01E-05 | 2.42E-05 | 1.67E-04 |
|  | **Standard deviation** | 4.07E-07 | 3.63E-06 | 1.18E-05 | 2.09E-03 | 2.46E-06 | 5.64E-06 | 4.21E-05 |
|  | **CV (%)** | 133.88 | 44.16 | 42.91 | 53.59 | 52.23 | 43.72 | 46.57 |
| **Reference Dose (*RfD*)** | | 1 | 1500 | 40 | 700 | 20 | 4 | 300 |

Table S13. Metal dose through dermal contact with particles contaminated by adults and children.

| **Target audience** | **Statistic** | **Cd** | | **Cr** | **Cu** | | **Fe** | | **Ni** | **Pb** | **Zn** |
| --- | --- | --- | --- | --- | --- | --- | --- | --- | --- | --- | --- |
|  |  | **μg kg^-1^ dia^-1^** | | | | | | | | | |
| **ADULT** | **Mean** | 4.16E-05 | | 1.12E-03 | 3.75E-03 | | 5.34E-01 | | 6.43E-04 | 1.76E-03 | 1.24E-02 |
|  | **Minimum** | 8.03E-07 | | 3.90E-04 | 1.28E-03 | | 9.12E-02 | | 2.10E-04 | 6.52E-04 | 2.30E-03 |
|  | **Maximum** | 1.81E-04 | | 2.36E-03 | 7.30E-03 | | 1.47E+00 | | 1.39E-03 | 3.31E-03 | 2.28E-02 |
|  | **Standard deviation** | 5.56E-05 | | 4.97E-04 | 1.61E-03 | | 2.86E-01 | | 3.36E-04 | 7.71E-04 | 5.76E-03 |
|  | **CV (%)** | 133.65 | | 44.38 | 42.93 | | 53.56 | | 52.26 | 43.81 | 46.45 |
| **CHILD** | **Mean** | 6.60E-05 | | 1.78E-03 | 5.95E-03 | | 8.46E-01 | | 1.02E-03 | 2.79E-03 | 1.96E-02 |
|  | **Minimum** | 1.27E-06 | | 6.19E-04 | 2.04E-03 | | 1.45E-01 | | 3.33E-04 | 1.03E-03 | 3.65E-03 |
|  | **Maximum** | 2.87E-04 | | 3.74E-03 | 1.16E-02 | | 2.33E+00 | | 2.20E-03 | 5.25E-03 | 3.62E-02 |
|  | **Standard deviation** | 8.82E-05 | 7.88E-04 | | 2.56E-03 | 4.53E-01 | | 5.32E-04 | | 1.22E-03 | 9.14E-03 |
|  | **CV (%)** | 133.64 | 44.27 | | 43.03 | 53.55 | | 52.16 | | 43.73 | 46.63 |
| **Reference Dose (*RfD*)** | | 1 | | 1500 | 40 | | 700 | | 20 | 4 | 300 |

Table S14. Available Consumption Rate (*CR*) of vegetables for adults and children.

|  | **Adult** | | | | | | | |
| --- | --- | --- | --- | --- | --- | --- | --- | --- |
| **Culture** | **Cd** | **Cr** | **Cu** | **Fe** | **Ni** | **Pb** | **Zn** | ***CR**** |
|  | **g day^-1^** | | | | | | |  |
| **Arugula** | 0.03 | 44.22 | 1.18 | 20.63 | 0.59 | 0.12 | 8.84 | **90** |
| **Iceberg lettuce** | 0.02 | 32.11 | 0.86 | 14.99 | 0.43 | 0.09 | 6.42 | **120** |
| **Red lettuce** | 0.04 | 67.02 | 1.79 | 31.28 | 0.89 | 0.18 | 13.40 | **120** |
| **Smooth lettuce** | 0.03 | 46.85 | 1.25 | 21.87 | 0.62 | 0.12 | 9.37 | **120** |
| **Curly lettuce** | 0.03 | 50.59 | 1.35 | 23.61 | 0.67 | 0.13 | 10.12 | **120** |
| **Spring onion** | 0.13 | 202.24 | 5.39 | 94.38 | 2.70 | 0.54 | 40.45 | **---** |
| **Radish** | 0.12 | 178.47 | 4.76 | 83.29 | 2.38 | 0.48 | 35.69 | **90** |
| **Aodai cucumber** | 0.11 | 167.93 | 4.48 | 78.37 | 2.24 | 0.45 | 33.59 | **130** |
| **Country cucumber** | 0.06 | 88.52 | 2.36 | 41.31 | 1.18 | 0.24 | 17.70 | **130** |
| **Mustard** | 0.27 | 402.10 | 10.72 | 187.65 | 5.36 | 1.07 | 80.42 | **60** |
|  | **Children** | | | | | | | |
| **Culture** | **Cd** | **Cr** | **Cu** | **Fe** | **Ni** | **Pb** | **Zn** | ****CR*** |
|  | **g day^-1^** | | | | | | | |
| **Arugula** | 0.01 | 15.35 | 0.41 | 7.16 | 0.2 | 0.04 | 3.07 | **90** |
| **Iceberg lettuce** | 0.01 | 11.15 | 0.3 | 5.2 | 0.15 | 0.03 | 2.23 | **120** |
| **Red lettuce** | 0.02 | 23.27 | 0.62 | 10.86 | 0.31 | 0.06 | 4.65 | **120** |
| **Smooth lettuce** | 0.01 | 16.27 | 0.43 | 7.59 | 0.22 | 0.04 | 3.25 | **120** |
| **Curly lettuce** | 0.01 | 17.56 | 0.47 | 8.2 | 0.23 | 0.05 | 3.51 | **120** |
| **Spring onion** | 0.05 | 70.22 | 1.87 | 32.77 | 0.94 | 0.19 | 14.04 | **---** |
| **Radish** | 0.04 | 61.97 | 1.65 | 28.92 | 0.83 | 0.17 | 12.39 | **90** |
| **Aodai cucumber** | 0.04 | 58.31 | 1.55 | 27.21 | 0.78 | 0.16 | 11.66 | **130** |
| **Country cucumber** | 0.02 | 30.74 | 0.82 | 14.34 | 0.41 | 0.08 | 6.15 | **130** |
| **Mustard** | 0.09 | 139.62 | 3.72 | 65.16 | 1.86 | 0.37 | 27.92 | **60** |

**CR*- Recommended Consumption by the Ministry of Health of Brazil (BRASIL, 2008);

--- Amount not informed.
